# Association of Oral Frailty with Physical Frailty and Malnutrition in Patients on Peritoneal Dialysis

**DOI:** 10.3390/nu17121950

**Published:** 2025-06-06

**Authors:** Yu Kobayashi, Tomomi Matsuoka, Ryo Yamaguchi, Kiyomi Ichijo, Miya Suzuki, Tomoyuki Saito, Kimihiro Igarashi, Tokiko Sato, Hiroyuki Takashima, Masanori Abe

**Affiliations:** Division of Nephrology, Hypertension and Endocrinology, Department of Medicine, Nihon University School of Medicine, Tokyo 173-8610, Japan; kobayashi.yu54@nihon-u.ac.jp (Y.K.); yamaguchi.ryo@nihon-u.ac.jp (R.Y.);

**Keywords:** malnutrition, oral frailty, peritoneal dialysis, physical frailty, sarcopenia

## Abstract

**Background**: Oral frailty is a state between normal oral function and oral hypofunction. Oral frailty progresses to oral hypofunction and dysphagia, which leads to malnutrition, and then to physical frailty and sarcopenia. Oral frailty is reported to be associated with physical frailty and malnutrition in hemodialysis patients, but there have been no reports on peritoneal dialysis (PD) patients. **Methods**: This prospective cohort study investigated the associations of oral frailty with physical frailty, sarcopenia, and malnutrition in patients on PD. Patients were divided into an oral frailty group and a non-oral frailty group according to the Oral Frailty Index-8. Patients were assessed for physical frailty, sarcopenia, and malnutrition at baseline and 1 year later, and changes in each measure were compared between the two groups. Physical frailty was assessed using the Revised Japanese version of the Cardiovascular Health Study Criteria (Revised J-CHS) and the FRAIL scale. Sarcopenia was assessed using the diagnostic criteria reported by the Asian Working Group for Sarcopenia in 2019 (AWGS2019 criteria) and the Screening Tool for Sarcopenia Combined with Calf Circumference (SARC-CalF), skeletal muscle index (SMI), calf circumference (CC), grip strength, and gait speed. Nutritional status was assessed with the Short-Form Mini-Nutritional Assessment (MNA-SF), the Malnutrition Universal Screening Tool (MUST), the Global Leadership Initiative on Malnutrition (GLIM) criteria, weight, and body mass index (BMI). **Results**: Of the 58 eligible patients, 51 completed the study. The oral frailty group was significantly older and had slower gait speed, fewer teeth, higher intact parathyroid hormone, higher C-reactive protein, higher frequency of cardiovascular disease, and lower employment at baseline. The oral frailty group had significantly worse physical frailty (Revised J-CHS, *p* = 0.047; FRAIL scale, *p* = 0.012), sarcopenia (SMI, *p* = 0.018; CC, *p* = 0.002), and nutritional status (MNA-SF, *p* = 0.029; MUST, *p* = 0.005; GLIM criteria, *p* = 0.022; weight, *p* < 0.001; BMI, *p* < 0.001). However, there were no significant differences in the worsening of sarcopenia (AWGS2019 criteria, SARC-CalF, grip strength, and gait speed). **Conclusions**: Oral frailty in patients on PD was associated with the development and progression of physical frailty and malnutrition, and may be associated with the development and progression of sarcopenia.

## 1. Introduction

Frailty has recently become a major issue as the population ages worldwide. As of September 2024, the population of elderly individuals aged ≥ 65 years in Japan is 36.25 million, accounting for 29.3% of the total population [1]; thus, Japan has become a super-aging society. Frailty is a state of increased vulnerability to stress due to a decrease in physiological reserve with age [2]. It is associated with adverse health outcomes including activity of daily living (ADL) disability, physical dysfunction, and poor prognosis [2,3]. Similarly, an association between frailty and adverse health outcomes has been reported in dialysis patients [4,5]. When detected early and appropriate interventions are provided, frailty is a reversible condition [6]. Preventing the development and progression of frailty in the elderly is important for improving their healthy lifespan and prognosis.

Sarcopenia is characterized by low levels of skeletal muscle mass with reduced strength and function. Frailty and sarcopenia interact with malnutrition. Malnutrition leads to loss of muscle mass and decreased activity, which in turn leads to the development and progression of physical frailty and sarcopenia. On the other hand, physical frailty and sarcopenia, through loss of muscle mass and decreased activity, cause weight loss and decreased appetite, exacerbating nutritional status [2]. This negative chain of events leads to adverse health outcomes, and the concept of “oral frailty” has recently been focused on as a contributing factor to this chain of events.

Oral frailty is a state between normal oral function and oral hypofunction [7]. Oral function in patients with oral frailty is impaired by deterioration of oral hygiene, age-related changes in oral function, a decrease in the number of teeth, decreased interest in oral hygiene, and decreased mental and physical reserve capacity. Therefore, oral frailty progresses to oral hypofunction and dysphagia, which leads to malnutrition and then to physical frailty and sarcopenia [8].

To prevent the development of oral frailty, personal and social risk factors are important in both public health and individual patient care. Personal risk factors for oral frailty include age, female sex, poor oral condition such as periodontal disease, tooth loss, xerostomia, reduced food diversity, poor sleep quality, and chronic illness [9,10,11]. Social risk factors for oral frailty have also been reported, including social isolation and isolated eating [9,12]. In Japan, where the population is aging, the number of elderly people living alone is increasing, and this is an important issue. In addition, polypharmacy is a risk factor for oral frailty, which needs to be fully understood by healthcare professionals [13]. Oral frailty has been reported to improve with appropriate interventions and treatments [14,15].

When chronic kidney disease (CKD) progresses to end-stage kidney disease (ESKD), renal replacement therapy (RRT) becomes necessary. RRT includes hemodialysis (HD), peritoneal dialysis (PD), and kidney transplantation. HD is a blood purification method in which blood is circulated outside the body through a dialyzer. In Japan, HD requires about 4 h of dialysis per session, 3 times per week, and patients must visit a dialysis facility, which limits their social activities. In addition, it is a rapid blood purification process that places a heavy burden on the body. On the other hand, PD is a blood purification method in which dialysate is placed in the peritoneal cavity and the peritoneum acts as a filter. Although PD requires 3 to 4 dialysate changes per day, it imposes fewer limits on social activities because dialysis can be performed at home or at work. It is also a relatively slow blood purification process that is less burdensome on the body. However, PD treatment results in a protein loss of 4 to 8 g/day [16].

Oral frailty in elderly non-dialysis patients is associated with physical frailty, sarcopenia, and mortality [17]. It has been reported that oral frailty is associated with physical frailty, sarcopenia, and malnutrition in CKD patients managed with non-dialysis and those managed with HD [18,19], but there have been no reports in PD patients. The aim of this study was to investigate the association between oral frailty and physical frailty, sarcopenia, and malnutrition in patients on PD.

## 2. Materials and Methods

### 2.1. Study Design and Setting

This was a prospective cohort study involving patients on PD at Nihon University Itabashi Hospital from 1 September 2023 to 31 March 2025. Patients were divided into oral frailty and non-oral frailty groups according to the presence of oral frailty at baseline. They were examined for various measures of physical frailty, sarcopenia, and nutritional status at baseline and 1 year later. The changes in each measure between the two groups were then compared. This study was conducted in accordance with the ethical principles of the Declaration of Helsinki and was approved by the Ethics Committee of Nihon University Itabashi Hospital. It was registered with the University Hospital Medical Information Network (UMIN000053222). All patients provided written informed consent.

### 2.2. Participants

The participants in this study were patients on PD at Nihon University Itabashi Hospital from 1 September 2023 to 31 March 2025. Inclusion criteria were age ≥ 20 years and ability to stand up. Because the measurement of the skeletal muscle index (SMI) requires standing for approximately 1 min, we defined a patient as able to stand if they could stand unaided for 1 min. The following exclusion criteria were applied: upper or lower limb defects, implanted medical devices such as cardiac pacemakers, transfer to exclusively HD, and refusal to participate in this study. Because this was a single-center study and the number of patients was limited, we included all patients who met the above criteria and gave consent to participate.

### 2.3. Assessment of Oral Frailty

Oral frailty was evaluated using the Oral Frailty Index-8 (OFI-8), which is a questionnaire to identify individuals at risk of oral frailty. The questionnaire consists of eight questions, with the total score ranging from 0 to 11 points. A total score of 0–2 points was defined as “low risk”, 3 points as “moderate risk”, and 4–11 points as “high risk” (Supplementary Table S1). In this study, patients with scores of 4–11 points were classified into the oral frailty group and those with scores of 0–3 points were classified into the non-oral frailty group [20].

### 2.4. Outcomes

#### Assessments of Physical Frailty, Sarcopenia, Nutritional Status

We used the following assessment tools to evaluate physical frailty, sarcopenia, and nutritional status. Physical frailty was assessed with the Revised Japanese version of the Cardiovascular Health Study Criteria (Revised J-CHS) and the FRAIL scale. Sarcopenia was assessed using the diagnostic criteria reported by the Asian Working Group for Sarcopenia in 2019 (AWGS2019 criteria) and the Screening Tool for Sarcopenia Combined with Calf Circumference (SARC-CalF). Nutritional status was assessed with the following tools: the Short-Form Mini-Nutritional Assessment (MNA-SF); the Malnutrition Universal Screening Tool (MUST); and the Global Leadership Initiative on Malnutrition (GLIM) criteria. We also used weight and body mass index (BMI) as assessments of nutritional status, and skeletal muscle index (SMI), calf circumference (CC), grip strength, and gait speed as assessments of sarcopenia. Low skeletal muscle mass was defined as SMI of <7.0 kg/m^2^ in men and <5.7 kg/m^2^ in women. The cutoff for CC was set to <34.0 cm in men and <33.0 cm in women. Low muscle strength was defined as grip strength of <28.0 kg in men and <18.0 kg in women. Low physical performance was defined as gait speed of <1.0 m/s. Fewer teeth was defined as having <20 teeth.

### 2.5. Assessment Tools

#### 2.5.1. Revised J-CHS

The Revised J-CHS is a modified version of the CHS criteria based on the phenotype model of Fried et al., which consists of the following five items: shrinking, weakness, exhaustion, slowness, and low activity. The scores range from 0 to 5 points, with 0–1 points for each component. A score of 0 is defined as “normal”, 1–2 points is defined as “poor”, and 3–5 points is defined as “frailty” (Supplementary Table S2) [21]. Muscle weakness was assessed by grip strength using a handgrip (Charder MG4800^®^, Charder Electronic, Co. Ltd., Taichung City, Taiwan). Grip strength was measured in a sitting position with the elbow joint flexed to 90°. It was measured with each hand, with the larger number used as the grip strength. Gait speed was assessed by a 6 m walk test. Speed was measured in the central 4 m but not in the 1 m before or after. The patients walked at a comfortable speed twice; the faster of the two measurements was used as the gait speed.

#### 2.5.2. FRAIL Scale

The FRAIL scale is a brief screening scale for physical frailty proposed by Morley et al. [22]. It consists of five questions on fatigue, resistance, ambulation, illnesses, and weight loss. The scores range from 0 to 5 points, with 0–1 points for each component. A score of 0 points is classified as “normal”, 1–2 points as “poor”, and 3–5 points as “frailty” (Supplementary Table S3) [22].

#### 2.5.3. AWGS2019 Criteria

The AWGS2019 criteria are used to diagnose sarcopenia in Asian populations and were reported by the AWGS in 2019. In the clinical setting, cases are extracted based on clinical symptoms or screening tests, and then skeletal muscle mass, muscle strength, and physical performance are evaluated in appropriate individuals. Low skeletal muscle mass and low muscle strength or low skeletal muscle mass and low physical performance are defined as “sarcopenia” (Supplementary Figure S1) [23]. In this study, all patients were undergoing PD for end-stage renal failure. Thus, they were considered eligible because they had clinical manifestations of chronic disease. Patients were evaluated for skeletal muscle mass, muscle strength, and physical performance at regular outpatient visits for PD. Patients on both PD and HD were evaluated for skeletal muscle mass after HD. Physical function was evaluated by a 6 m walk test. Gait speed < 1.0 m/s was defined as low physical performance according to the AWGS2019 criteria. Grip strength and 6 m walk test were evaluated in the same way as in the Revised J-CHS. Skeletal muscle mass was evaluated by SMI using multifrequency bioelectrical impedance analysis (InBody 270^®^ body analyzer, InBody, Tokyo, Japan). SMI was measured without intraperitoneal dialysate fluid in the patients, and was calculated using the appendicular skeletal muscle mass (ASM) and height by the following formula:

SMI = ASM (kg)/height (m)^2^

#### 2.5.4. SARC-CalF

The SARC-CalF is a screening tool that adds an assessment of CC to the SARC-F. The SARC-F consists of five questions on strength, assistance while walking, rising from a chair, climbing stairs, and falls. The scores range from 0 to 10 points, with 0–2 points for each component [24]. When the CC is below the cutoff value of 34.0 cm in men and 33.0 cm in women, 10 points are added to the score. The score ranges from 0 to 20 points, with a score of 11–20 points indicating a high probability of sarcopenia (Supplementary Table S4) [25]. In this study, CC was measured at the thickest point of the lower leg. It was measured on both legs; the thinner leg was used as the CC.

#### 2.5.5. MNA-SF

The MNA-SF is a nutritional screening tool proposed by Rubenstein et al. [26]. The MNA-SF consists of the following five components: declined food intake, weight loss, mobility, psychological stress or acute disease, neuropsychological problems, and BMI or CC. The scores range from 0 to 14 points, with 0–2 or 0–3 points for each component. A score of 0–7 points is defined as “malnourished”, 8–11 points is defined as “at risk of malnutrition”, and 12–14 points is defined as “normal nutritional status” (Supplementary Table S5) [26].

#### 2.5.6. MUST

MUST is a nutritional screening tool for adults proposed by the British Association for Parenteral and Enteral Nutrition and consists of three components: BMI, weight loss, and acute illness with no nutritional intake. The scores ranged from 0 to 6 points, with 0–2 points for each component. The risk of malnutrition was defined as low (0 points), medium (1 point), and high (2–6 points) (Supplementary Figure S2) [27].

#### 2.5.7. GLIM Criteria

The GLIM criteria are the diagnostic criteria for malnutrition proposed by the world’s leading clinical nutrition societies in 2018. The criteria screen for malnutrition, diagnose malnutrition, and determine severity. Screening for malnutrition is performed by validated nutrition screening tools, such as the MUST, MNA-SF, and Subjective Global Assessment. The diagnosis of malnutrition is divided into phenotypic and etiologic criteria. Phenotypic criteria consist of weight loss, low BMI, and reduced muscle mass, while the etiological criteria consist of reduced food intake or assimilation and disease burden/inflammation. Malnutrition is diagnosed when at least one item in each criterion is met. If malnutrition is diagnosed and at least one of the phenotypic criteria highly exceeds the reference value, the case is classified as severe malnutrition; other cases are classified as moderate malnutrition (Supplementary Figure S3) [28]. In this study, MNA-SF was used as a screening tool for malnutrition. All patients were undergoing PD for ESKD and therefore met the disease burden/inflammation definition in the etiologic criteria. As in the AWGS2019 criteria, muscle mass was evaluated by SMI using multifrequency bioelectrical impedance analysis (InBody 270^®^ body analyzer, InBody, Tokyo, Japan). Subjects were also compared between the two groups for the presence of malnutrition.

### 2.6. Data Collection

Demographic data such as height, body weight, BMI, automated peritoneal dialysis/continuous ambulatory peritoneal dialysis (APD/CAPD), dialysate volume, number of dialysate exchanges, cause of ESKD, comorbidities (diabetes mellitus, hypertension, hyperlipidemia, cardiovascular disease (CVD), and PD-related peritonitis), drinking, and smoking were collected from the medical records. APD included nightly PD, continuous cyclic PD type I (CCPD I), continuous cyclic PD type II (CCPD II), and tidal PD. The dialysate volume per day (mL) was defined as the sum of dextrose and icodextrin solutions. The number of dialysate exchanges was defined as the number of exchanges including dextrose and icodextrin solutions.

Laboratory data from the most recent evaluation were also collected from medical records. An automatic analyzer (LABOSPECT^®^ 008α, Hitachi High-Tech Corporation, Tokyo, Japan) was used to perform the following serum biochemistry tests as described previously [19]. Serum urea nitrogen was measured using ultraviolet absorption spectrophotometry. Serum creatinine was measured using an enzymatic assay. Serum albumin was measured using the revised bromocresol purple assay. Serum sodium, potassium, and chloride were measured using an ion-selective electrode assay. Total cholesterol was measured using the cholesterol oxidase and peroxidase enzymatic assay. Total iron-binding capacity (TIBC) was measured using the colorimetric method. Intact parathyroid hormone was measured using an electrochemiluminescence immunoassay. Hemoglobin was measured using the sodium lauryl sulphate-hemoglobin detection assay. Serum calcium was measured by an enzymatic assay using the alpha-amylase reaction. Serum phosphate was measured using an enzymatic reaction. C-reactive protein (CRP) levels were measured using the latex agglutination immunoassay.

Weight, BMI, and SMI were measured without intraperitoneal dialysate fluid in the patients. This study included patients on PD + once-weekly HD combination therapy. For patients on once-weekly HD, clinical dry weight was used for the weight and BMI, and their SMI was measured after the completion of HD.

### 2.7. Statistical Analysis

Continuous variables were expressed as the mean ± standard deviation or median (interquartile range), and categorical variables were expressed as the number (percentage). The normality of the data was assessed using normal quantile plots. First, patient characteristics and various assessment tools of physical frailty, sarcopenia, and nutritional status at baseline were compared between the oral frailty group and the non-oral frailty group. Continuous variables were compared using Student’s *t*-test, Welch’s *t*-test, or the Wilcoxon rank sum test, and categorical variables were compared using the chi-square test or Fisher’s exact test, as appropriate. One-year changes in body indicators such as SMI, CC, grip strength, gait speed, weight, and BMI were compared between the two groups using Student’s *t*-test. To assess the adjusted association between oral frailty at baseline and 1-year changes in body indicators, general linear models (GLMs) were used. Each model included the 1-year change in a given indicator as the dependent variable and the OFI-8 risk category as the main independent variable. We adjusted for four baseline covariates: age, CRP, history of CVD, and employment. These covariates were selected based on both clinical relevance and statistically significant differences between groups observed at baseline. Third, the 1-year changes in total scores for the assessment tools were compared. The changes in total scores for each assessment tool were calculated and categorized as “improvement or no change” or “worsening”. However, because the AWGS2019 criteria and the GLIM criteria were not scored, the changes in these risk categories were determined. These changes in total scores or risk categories between the two groups were compared using the chi-square test or Fisher’s exact test. Furthermore, we investigated factors potentially associated with the prevalence of oral frailty. Univariate logistic regression analysis was performed using the OFI-8 risk category as the dependent variable and clinical variables as independent variables. Multivariate logistic regression analysis was then performed to identify risk factors for oral frailty. Variables with a *p* value of <0.05 in the univariate analysis were included in the multivariate model, except for the number of remaining teeth, which was excluded due to its conceptual and clinical overlap with the definition of oral frailty. Stepwise variable selection was performed using the minimum Akaike information criterion as the stopping criterion, following a backward elimination approach. Statistical analyses were performed with JMP^®^ Pro ver. 17.2 (SAS Institute Inc., Cary, NC, USA) and the R^Ⓡ^ver. 4.4.3 (R Foundation for Statistical Computing, Vienna, Austria). *p* values of < 0.05 were considered statistically significant.

## 3. Results

### 3.1. Prevalence of Oral Frailty

**Figure 1:**
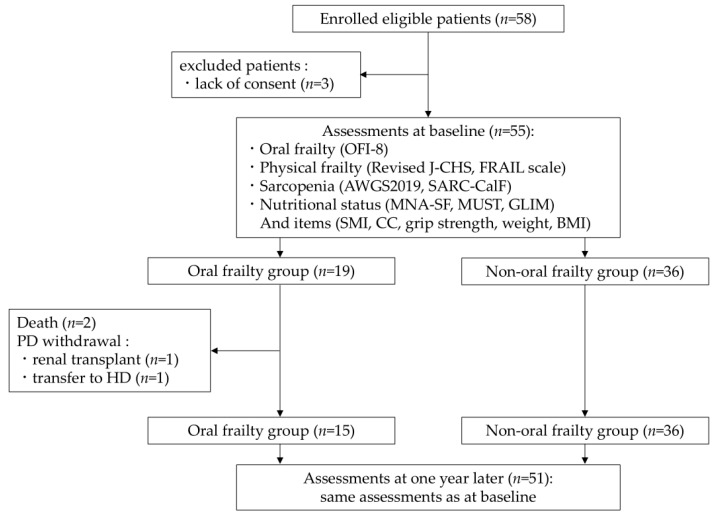
Flow diagram of patients in the study. AWGS2019, Asian Working Group for Sarcopenia in 2019; BMI, body mass index; CC, calf circumference; GLIM, Global Leadership Initiative on Malnutrition criteria; HD, hemodialysis; J-CHS, Japanese version of the Cardiovascular Health Study; MNA-SF, Short-Form Mini-Nutritional Assessment; MUST, Malnutrition Universal Screening Tool; OFI-8, Oral Frailty Index-8; PD, peritoneal dialysis; SARC-CalF, Screening Tool for Sarcopenia Combined with Calf Circumference; SMI, skeletal muscle index.

**Figure 2:**
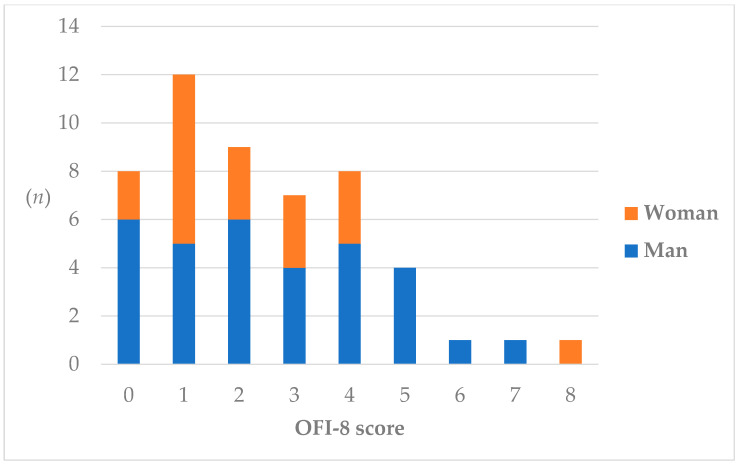
Distribution of OFI-8 scores. Patients with scores of ≤3 were classified into the non-oral frailty group, while those with scores of ≥4 were classified into the oral frailty group. OFI-8, Oral Frailty Index-8.

A flowchart of patients in this study is shown in Figure 1. In total, 58 eligible patients were enrolled. Five patients were excluded due to lack of consent (*n* = 3), PD withdrawal due to renal transplant (*n* = 1) or transfer to HD (*n* = 1). Two of the remaining fifty-three patients died during the 1-year follow-up period. The final remaining 51 patients (32 men, 19 women; mean age, 59.1 ± 12.8 years) were examined for baseline OFI-8. The percentages of OFI-8 scores are shown in Figure 2. In total, 15 patients (11 men, 4 women; mean age, 66.0 ± 14.2 years) had oral frailty, accounting for 29.4% of the total.

#### 3.1.1. Clinical Characteristics at Baseline

The clinical characteristics of the study are shown in Table 1. There were significant differences between the non-oral frailty group and the oral frailty group in age, gait speed, number of teeth, diastolic blood pressure, heart rate, intact PTH, CRP, frequency of CVD, and employment. There were no significant differences in sex, duration of PD, height, weight, BMI, SMI, CC, grip strength, systolic blood pressure, serum urea nitrogen, creatinine, hemoglobin, albumin, corrected calcium, phosphate, TIBC, total cholesterol, use of APD, dialysate volume, HD combination, cause of ESKD, PD-related peritonitis, smoking, drinking, or comorbidities such as diabetes mellitus, hypertension, and dyslipidemia.

To identify factors associated with oral frailty, univariate logistic regression analysis was performed using the OFI-8 risk category as the dependent variable and the clinical variables in all the patients (Table 2). Oral frailty was significantly associated with age, gait speed, fewer teeth, intact PTH, CRP, history of CVD, and unemployment. A multivariate logistic regression analysis was performed using backward stepwise elimination (Table 2). CRP and history of CVD was significantly associated with oral frailty in the adjusted model. However, there was no significant association between oral frailty and unemployment.

#### 3.1.2. Risk Categories for Assessments of Physical Frailty, Sarcopenia, and Nutritional Status at Baseline

There were significant differences between the two groups in the risk categories for the Revised J-CHS and FRAIL scale as assessments of physical frailty and the SARC-CalF as an assessment of sarcopenia. However, there were no significant differences in the risk categories for the AWGS2019 criteria as an assessment of sarcopenia and the MNA-SF, MUST, and GLIM criteria as assessments of nutritional status (Table 3).

#### 3.1.3. One-Year Changes in Body Indicators

There were significant differences between the non-oral frailty group and the oral frailty group in the changes in SMI and CC as assessments of sarcopenia, and weight and BMI as assessments of nutritional status over a 1-year period. However, there was no significant difference in grip strength and gait speed as assessments of sarcopenia (Table 4).

Table 5 summarizes the results of using GLMs to evaluate the association between the OFI-8 risk category and 1-year changes in body indicators. After adjusting for age, CRP, history of CVD, and employment, there were significant differences between the non-oral frailty group and the oral frailty group in the 1-year changes in weight and BMI. However, there were no statistically significant differences in the 1-year changes in SMI, CC, grip strength, and gait speed.

#### 3.1.4. One-Year Changes in Risk Categories and Total Scores for Assessments of Physical Frailty, Sarcopenia, and Nutritional Status

**Figure 3:**
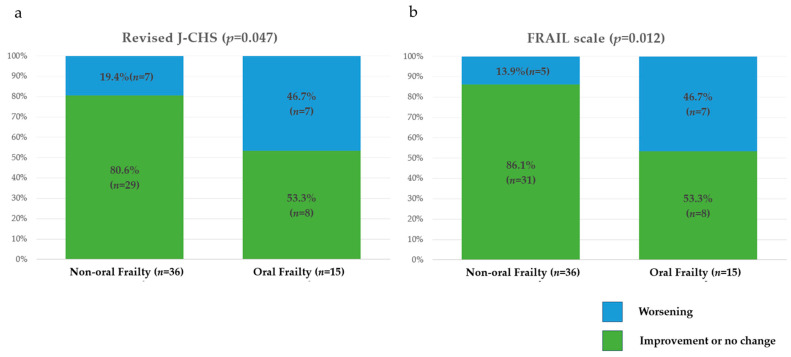
One-year changes in the risk categories and total scores for assessments of physical frailty in the two groups. (**a**) Revised J-CHS; (**b**) FRAIL scale. J-CHS, Japanese version of the Cardiovascular Health Study.

**Figure 4:**
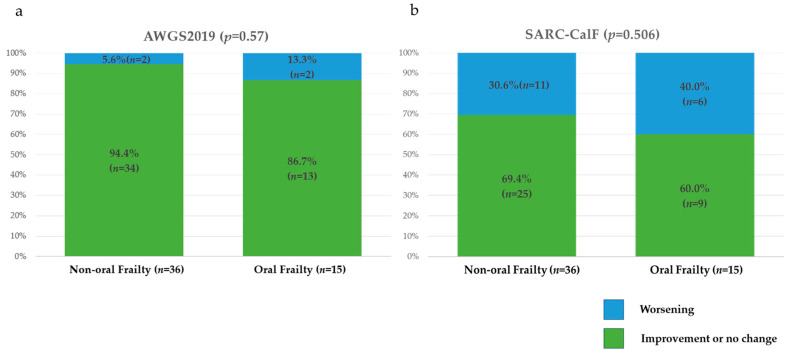
One-year changes in the risk categories and total scores for assessments of sarcopenia in the two groups. (**a**) AWGS2019; (**b**) SARC-CalF AWGS2019, Asian Working Group for Sarcopenia in 2019; SARC-CalF, Screening Tool for Sarcopenia Combined with Calf Circumference.

**Figure 5:**
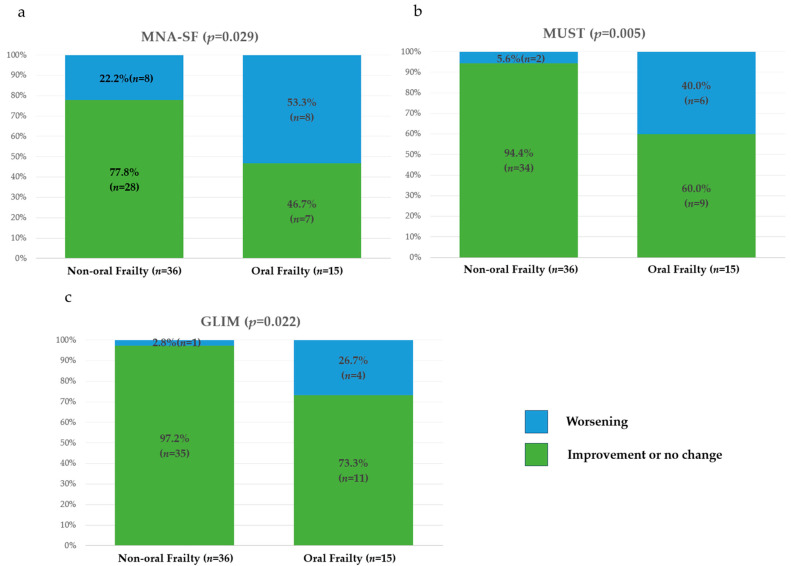
One-year changes in the risk categories and total scores for assessments of nutritional status in the two groups. (**a**) MNA-SF; (**b**) MUST; (**c**) GLIM. GLIM, Global Leadership Initiative on Malnutrition; MNA-SF, Short-Form Mini-Nutritional Assessment; MUST, Malnutrition Universal Screening Tool.

There were significant differences between the two groups in the deterioration of total scores of the Revised J-CHS and FRAIL scale as assessments of physical frailty over a 1-year period (Figure 3). There were no significant differences in the deterioration of total scores of the SARC-CalF or AWGS2019 risk category as assessments of sarcopenia over a 1-year period (Figure 4). There were significant differences in the deterioration of total scores of the MNA-SF, MUST, and GLIM risk category as assessments of nutritional status over a 1-year period (Figure 5).

## 4. Discussion

This study showed that oral frailty was associated with the development and progression of physical frailty and malnutrition in patients on PD for the first time. The oral frailty group was significantly older and had fewer teeth, slower gait speed, lower diastolic blood pressure, lower heart rate, higher intact PTH, higher CRP, higher frequency of CVD, and fewer workers. In addition, CRP and history of CVD were independent predictors of oral frailty in the adjusted model. Furthermore, the oral frailty group had significantly worse ratings on the Revised J-CHS, FRAIL scale, and SARC-CalF, suggesting a higher risk of physical frailty and sarcopenia. After 1 year, the body indicators SMI, CC, weight, and BMI significantly decreased in patients on PD in the oral frailty group. In the adjusted model, weight and BMI significantly decreased. These findings suggest that oral frailty may be predictive of nutritional deterioration over time, particularly with respect to body mass and adiposity, rather than muscle strength. In addition, the total scores of the Revised J-CHS, FRAIL scale, MNA-SF, MUST, and GLIM risk category were significantly worse in patients on PD in the oral frailty group, suggesting the development and progression of physical frailty and malnutrition.

Oral frailty is a state between normal oral function and oral hypofunction, and it is a factor that causes physical frailty [7]. Failure to appropriately intervene for minor oral deterioration and decreased interest in oral health can lead to poor oral function and swallowing dysfunction, which in turn can lead to malnutrition and low physical and mental function [8]. Tanaka et al. proposed that oral frailty should be diagnosed if at least three of the following six items are present: (1) <20 natural teeth, (2) decreased chewing ability, (3) decreased oral motor skill, (4) decreased tongue pressure, (5) difficulties eating tough foods and (6) difficulties in swallowing tea or soup [17]. In this study, oral frailty was assessed using the OFI-8, which is a questionnaire consisting of eight subjective items. It does not include objective dental assessment measures, but includes items on decreased interest in oral health and decreased social activity. It has already been reported that OFI-8 adequately screens for oral frailty [20]. Also, OFI-8 has been reported to be a useful prognostic tool in the general elderly population [29]. In Japan, it is not feasible in practice for all dialysis patients to visit a dental clinic. Of the dialysis patients in Japan, 82.9% received hemodialysis at medical facilities without dental departments [30]. Also, only 19.2% of small clinics without a dental department had a cooperative registered dental clinic. Dialysis facilities without dental departments tended to be more reluctant to collaborate in dental care [30]. Dialysis patients are reluctant to visit other departments, including dentistry, because of the time required for dialysis therapy and hospital visits; therefore, a simple and appropriate test to evaluate oral frailty during dialysis or as an outpatient is desirable [19]. Similarly, the OFI-8 was used to assess oral frailty in the studies of patients on HD [19,31]. The number of teeth at baseline was lower in the oral frailty group than in the non-oral frailty group. In multivariate logistic regression analysis, fewer teeth was an independent predictor of oral frailty. Maintenance dialysis patients in Japan have significantly fewer remaining teeth and more denture users in comparison with the general elderly population [32]. The ORAL-D study reported that missing or filled teeth are associated with early mortality in patients on HD [33]. Some oral frailty assessments include the number of teeth as a subitem [17,34]. Number of teeth is relatively easy to measure even in dialysis facilities with limited dental collaboration. It may be useful to use the number of teeth in addition to the OFI-8 as an assessment of oral frailty.

The prevalence of oral frailty was 29.4% of the total in the study. The prevalence of oral frailty in the general elderly population has been reported to range from 16.0% to 44.3% [20,35,36], but each study used different age groups and assessment methods. The prevalence of oral frailty, as determined by the OFI-8, tends to be high [35]. The prevalence of oral frailty in patients on HD has been reported to range from 38.8% to 61.2% [19,31,37], which is higher than in non-dialysis patients. To our knowledge, the prevalence of oral frailty in patients on PD has not been reported. The prevalence of oral frailty was lower in this study than in studies of patients on HD. This may be attributable to the lower average age and higher social activity of the patients in the study. In Japan, PD is often the RRT of choice for socially active patients. According to the JSDT Renal Data Registry, the mean age of patients on PD in Japan is 64.66 ± 14.84, which is approximately 5 years younger than the mean age of patients on HD [38].

The oral frailty group had significantly higher intact PTH, higher CRP, and a higher frequency of CVD. Poor oral hygiene can lead to the development of periodontal disease and dental caries, leading to a chronic inflammatory state, and it is consequently a risk factor for physical frailty and sarcopenia [39]. Oral frailty could lead to the development of CVD [40]. On the other hand, chronic diseases have been reported to exacerbate anorexia [41], and periodontal disease is reported to be more common and severe in dialysis patients [42]. Also, it has been reported that PTH is involved in adipose tissue browning and muscle atrophy due to increased energy expenditure [43]. Elevated intact PTH is an indicator of hyperphosphatemia in CKD. A series of mechanisms for secondary hyperparathyroidism in CKD are associated with risk of CVD, fractures, and increased mortality [44]. In addition, it has been reported from the Dialysis Outcomes and Practice Patterns Study that secondary hyperparathyroidism in patients on HD is associated with weight loss, and that weight loss is associated with increased long-term mortality [45]. However, it has been reported that restricted dietary phosphate reduced sarcopenia in old mice [46]. Future studies are needed to investigate the relationship between interventions for secondary hyperparathyroidism and frailty and sarcopenia in dialysis patients.

The prevalence of physical frailty in elderly community-dwelling people has been reported to range from 6.9% to 16.3% [3,47]. An observational study of 542 patients on HD in Japan investigated the prevalence of physical frailty using the Revised J-CHS, reporting a prevalence of 21.4% for physical frailty and 52.6% for pre-frailty [48]. The prevalence of physical frailty in PD patients has been reported to range from 19.3% to 41% [49,50]. In the present study, the prevalence of physical frailty and pre-frailty at baseline was 27.5% and 51%, respectively, which is consistent with previous reports. The study suggested that oral frailty is associated with physical frailty, and that oral frailty progresses to physical frailty. The prevalence of physical frailty with oral frailty was very high, at 60%, in this study. In the study of patients on HD, the prevalence of physical frailty with oral frailty has been reported to be 44.9% [19]. Dialysis patients with oral frailty could have a high risk of developing physical frailty. There were no statistically significant differences in weight and BMI at baseline between the two groups, but weight and BMI were significantly decreased in the oral frailty group over a 1-year period. Makizako et al. reported that slowness, weakness, and weight loss, among the CHS criteria based on the phenotype model reported by Fried et al., were associated with the risk of developing functional disability [51]. Ku et al. reported that weight loss of ≥5% per year in patients with CKD was associated with a significantly higher risk of death after the initiation of dialysis [52]. Oral frailty in patients on PD might lead to weight loss and progressive physical frailty, thereby increasing the risk of developing functional disability and mortality.

The SARC-CalF risk category at baseline was significantly worse in the oral frailty group than in the non-oral frailty group. The reason for using the SARC-CalF to assess sarcopenia is that the SARC-CalF and CC are reported to reflect sarcopenia better than the SARC-F in patients on PD [53]. Furthermore, the SARC-CalF predicts mortality in dialysis patients better than the SARC-F [54]. Therefore, the SARC-CalF seems to be an appropriate tool for predicting sarcopenia and life expectancy in dialysis patients. In this study, SMI and CC were not significantly different between the two groups at baseline, but they were significantly lower in the oral frailty group than in the non-oral frailty group over a 1-year period. It has been reported that a declining level of muscle mass is associated with all-cause mortality in patients on PD [55]. It has also been reported that CC has a stronger association with SMI and muscle strength than other screening tools in patients on PD [53]. In dialysis facilities where SMI cannot be measured, the measurement of CC is an easily used indicator to predict sarcopenia. These results suggested that oral frailty in patients on PD could be associated with sarcopenia. It has been reported that oral frailty in patients on HD is significantly associated with the development and progression of sarcopenia [19]. Further studies are needed to investigate oral frailty in patients on PD with a larger number of patients and over a longer period of time.

Malnutrition is associated with mortality in patients on maintenance dialysis [56,57]. We used the MNA-SF, MUST, and GLIM criteria to assess malnutrition in this study. The usefulness of these assessments in patients on PD has been reported. It has been reported that the MNA-SF is associated with all-cause mortality in maintenance dialysis patients [58]. The GLIM is used worldwide as a diagnostic criterion for malnutrition and has been reported to better reflect malnutrition [59]. But it has been reported that the GLIM has reduced sensitivity in patients on HD [60]. On the other hand, it has been reported that the GLIM is associated with mortality in PD patients [61]. Wang et al. reported that a group of PD patients diagnosed as malnourished based on the GLIM had poor prognosis, with an adjusted all-cause mortality hazard ratio of 2.91 at a median follow-up of 45 months [61]. To our knowledge, no studies on the MUST in patients on PD have been reported. However, it has been reported that MUST is associated with mortality and prolonged hospitalization in the elderly [62]. MUST is designated as an appropriate screening tool in the GLIM [28]. In this study, there was no association between oral frailty and any of the nutritional assessment tools used at baseline. Thus, there was no difference in nutritional status at baseline between the oral frailty group and the non-oral frailty group. However, the MNA-SF, MUST, and GLIM criteria significantly worsened in the oral frailty group over a 1-year period. Also, oral frailty was significantly associated with weight loss and decreased BMI in the adjusted model. Weight loss and decreased BMI are used as subitems in many assessment tools of nutritional status and are among the items most reflective of malnutrition [28]. Thus, this study showed that oral frailty is associated with the development and progression of malnutrition.

There are several reports of early intervention for oral frailty resulting in improved oral or general health status. A cluster-randomized controlled trial showed that oral diadochokinesis and tongue pressure were significantly improved in patients with oral frailty after a 12-week oral frailty intervention program [14]. In those patients, tongue lifting exercise for 3 months significantly improved oral function, including tongue pressure and oral diadochokinesis, and physical functions, including open-eyed one-leg standing time and sit-to-stand motion, in both frailty and robust groups. That trial found that changes in body composition, such as decreases in visceral fat level and basal metabolic rate, did not significantly change in the frailty group, but did significantly change in the robust group [63]. These results suggest that it is important to intervene in oral frailty before the progression of frailty and sarcopenia in the elderly. A program in which participants listened to lectures on oral hygiene, nutrition, and diet while eating a lunch containing foods that strengthen chewing ability has been reported to result in significant improvements in the level and severity of oral frailty [64]. Oral frailty in dialysis patients may be associated with adverse health outcomes and requires early detection and intervention. To prevent oral frailty, it may be important to have regular dental checkups, manage CVD appropriately, and stay active, including employment. If interventions for oral frailty can reduce the development and progression of physical frailty, sarcopenia, and malnutrition, it may reduce the incidence of CVD and infectious diseases, and even reduce the cost of hospitalization and medical care. Therefore, further studies are needed to verify the effectiveness of interventions for oral frailty in dialysis patients.

This study has several limitations. First, the follow-up period of this study was 1 year, which may not have been long enough to show significant worsening of sarcopenia. Longer-term observational studies are needed to investigate mortality. Second, we measured skeletal muscle mass to assess sarcopenia, but due to the limitation of the facility’s equipment, we measured it only in patients who were able to stand; those who were unable to stand were excluded. Third, we used the OFI-8 to assess oral frailty, which consists of a subjective questionnaire and does not include objective measures of the oral status, masticatory function, or swallowing function performed by a dentist. Oral frailty may be currently underdiagnosed. However, OFI-8 has been reported to have a sensitivity of 80% and specificity of 80% for the diagnosis of oral frailty [20]. Therefore, if large-scale screening by a simple method is performed, then there could be a major impact. Fourth, patients on PD were included in this study, although some patients were on both PD and HD. It has been reported that PD + HD combination therapy improves uremic symptoms and increases quality of life [65]. However, in Japan, 20% of patients on PD are receiving PD + HD combination therapy [38], which is becoming popular. The rate of PD + HD combination therapy in this study was 15.7%. Fifth, the oral frailty group were older and had fewer teeth, a higher frequency of CVD, and lower employment compared to the non-oral frailty group. Additionally, the oral frailty group was dominated by males, and sex differences were found in OFI-8 scores. The result of this study may be partly attributed to selection bias in the control group due to the small sample size. In addition, the small sample size may have resulted in low statistical power. Therefore, further studies with an increased sample size and propensity score matching are needed.

## 5. Conclusions

This study showed that oral frailty in patients on PD predicts the development and progression of physical frailty and malnutrition. Early assessment and appropriate intervention for oral frailty in patients on PD are expected to prevent the development and progression of physical frailty and malnutrition.

## Figures and Tables

**Table 1:** Clinical characteristics.

Variable	All Patients	Non-Oral Frailty Group	Oral Frailty Group	*p* Value
Patients, *n* (male, %)	51 (62.8)	36 (58.3)	15 (73.3)	0.313
Age, years	59.1 ± 12.8	56.1 ± 10.8	66.3 ± 14.7	0.009
Duration of dialysis, months	60 (34–147)	56 (25–141)	63 (44–171)	0.380
Height, m	1.65 ± 0.07	1.66 ± 0.07	1.64 ± 0.08	0.396
Weight, kg	65.3 ± 14.5	64.9 ± 14.1	66.2 ± 15.9	0.772
BMI, kg/m^2^	23.8 ± 4.4	23.5 ± 4.1	24.6 ± 5.1	0.440
SMI, kg/m^2^	7.7 ± 1.5	7.8 ± 1.7	7.5 ± 0.9	0.547
CC, cm	35.5 ± 4.4	35.8 ± 4.0	35.0 ± 5.4	0.574
Grip strength, kg	25.9 ± 8.5	26.5 ± 7.7	24.6 ± 10.5	0.487
Gait speed, m/s	1.1 (0.8–1.2)	1.1 (1.0–1.2)	0.8 (0.7–1.1)	<0.001
Number of teeth, <20 (%)	8 (15.7)	2 (5.6)	6 (40.0)	0.005
Systolic blood pressure, mmHg	140 ± 16	142 ± 17	137 ± 15	0.389
Diastolic blood pressure, mmHg	82 ± 12	85 ± 10	74 ± 11	0.002
Heart rate, bpm	82 ± 14	85 ± 14	75 ± 14	0.031
Serum urea nitrogen, mg/dL	52.3 ± 12.1	52.6 ± 12.7	51.6 ± 10.8	0.780
Creatinine, mg/dL	9.6 ± 3.4	10.0 ± 3.7	8.8 ± 2.7	0.247
Hemoglobin, g/dL	11.0 ± 1.1	11.0 ± 1.2	11.3 ± 0.8	0.251
Albumin, g/dL	3.2 ± 0.5	3.3 ± 0.5	3.0 ± 0.5	0.090
Corrected calcium, mg/dL	9.1 ± 0.7	9.1 ± 0.8	9.0 ± 0.5	0.468
Phosphate, mg/dL	5.4 ± 1.4	5.4 ± 1.3	5.4 ± 1.6	0.907
Intact PTH, pg/mL	212 (148–377)	183 (132–297)	275 (198–631)	0.045
CRP, mg/dL	0.11 (0.1–0.48)	0.1 (0.1–0.29)	0.48 (0.2–1.73)	0.011
TIBC, µg/dL	256 ± 58	263 ± 62	240 ± 46	0.206
Total cholesterol, mg/dL	174 ± 37	179 ± 39	160 ± 25	0.090
APD/CAPD, (*n*)	44/7	32/3	12/4	0.174
Dialysate volume, mL/day	6271 ± 2425	6428 ± 2352	5893 ± 2637	0.479
Number of dialysate exchanges, times/day	4 (3–5)	4 (3–5)	4 (3–4)	0.492
HD combination, *n* (%)	8 (15.7)	4 (11.1)	4 (26.7)	0.213
Cause of ESKD, *n* (%)				
Diabetic nephropathy	21	14 (27.5)	7 (13.7)	0.607
Hypertension	13	8 (15.7)	5 (9.8)	0.407
Chronic glomerular nephritis	2	2 (3.9)	0 (0)	1.000
IgA nephropathy	5	4 (7.8)	1 (2.0)	1.000
Polycystic kidney disease	6	5 (9.8)	1 (2.0)	0.657
Others	4	3 (5.9)	1 (2.0)	1.000
Comorbidities, *n* (%)				
Diabetes mellitus	23 (45.1)	15 (41.7)	8 (53.3)	0.446
Hypertension	50 (98.0)	35 (97.2)	15 (100)	1.000
Dyslipidemia	33 (64.7)	22 (61.1)	11 (73.3	0.527
CVD	12 (23.5)	3 (8.3)	9 (60.3)	<0.001
PD-related peritonitis	24 (47.1)	17 (47.2)	7 (46.7)	0.971
Smoking, *n* (%)	27(52.9)	17 (47.2)	10 (66.7)	0.205
Drinking, *n*(%)	15 (29.4)	10 (27.8)	5 (33.3)	0.692
Employment, *n* (%)	31 (60.8)	27 (75.0)	4 (26.7)	0.002

APD, automated peritoneal dialysis; BMI, body mass index; CAPD, continuous ambulatory peritoneal dialysis; CC, calf circumference; CRP, C-reactive protein; CVD, cardiovascular disease; ESKD, end-stage kidney disease; HD, hemodialysis; PD, peritoneal dialysis; PTH, parathyroid hormone; SMI, skeletal muscle index; TIBC, total iron-binding capacity.

**Table 2:** Univariate and multivariate logistic regression analyses of risk factors for oral frailty.

Variables	Univariate				Multivariate (R^2^ = 0.495)			
	OR	95% CI		*p* Value	OR	95% CI		*p* Value
		Lower	Upper			Lower	Upper	
Male sex (vs. female)	1.96	0.52	7.37	0.317				
Age (per 1-year increase)	1.07	1.02	1.14	0.016				
Duration of dialysis (per month)	1.00	0.99	1.01	0.398				
BMI (per 1 kg/m^2^ increase)	1.06	0.92	1.21	0.433				
SMI (per 1 kg/m^2^ increase)	0.87	0.57	1.34	0.540				
CC (per 1 cm increase)	0.96	0.84	1.04	0.566				
Grip strength (per 1 kg increase)	0.97	0.91	1.03	0.479				
Gait speed (per 1 m/s increase)	0.01	0.0004	0.20	0.003				
Fewer teeth (<20 teeth vs. ≥20 teeth)	11.3	1.95	65.9	0.007				
Serum urea nitrogen (per 1 mg/dL increase)	0.99	0.94	1.01	0.775				
Creatinine (per 1 mg/dL increase)	0.90	0.75	1.08	0.243				
Hemoglobin (per 1 g/dL increase)	1.43	0.78	2.60	0.249				
Albumin (per 1 g/dL increase)	0.31	0.08	3.23	0.095				
Corrected calcium (per 1 mg/dL increase)	0.70	0.28	1.80	0.462				
Phosphate (per 1 mg/dL increase)	1.03	0.66	1.60	0.905				
Intact PTH (per 1 pg/dL increase)	1.00	1.00	1.01	0.022				
CRP (per 1 mg/dL increase)	7.96	1.88	33.8	0.005	12.2	2.01	74.2	0.007
TIBC (per 1 µg/dL increase)	0.99	0.98	1.01	0.208				
Total cholesterol (per 1 mg/dL increase)	0.98	0.97	1.00	0.093				
APD (vs. CAPD)	0.25	0.05	1.30	0.099				
Diabetes mellitus (yes vs. no)	1.23	0.37	4.12	0.743				
Hyperlipidemia (yes vs. no)	1.75	0.47	6.59	0.408				
History of CVD (yes vs. no)	16.5	3.43	79.3	<0.001	16.6	2.17	127.6	0.007
History of PD-related peritonitis (yes vs. no)	0.98	0.29	3.27	0.971				
Smoking (current smoker vs. non-smoker)	2.24	0.64	7.86	0.21				
Drinking (yes vs. no)	1.30	0.36	4.76	0.692				
Unemployment (yes vs. employed)	8.25	2.10	32.5	0.003	5.82	0.87	39.0	0.069

APD, automated peritoneal dialysis; BMI, body mass index; CAPD, continuous ambulatory peritoneal dialysis; CC, calf circumference; CRP, C-reactive protein; CVD, cardiovascular disease; PD, peritoneal dialysis; PTH, parathyroid hormone; SMI, skeletal muscle index; TIBC, total iron-binding capacity.

**Table 3:** Assessments of physical frailty, sarcopenia, and nutritional status at baseline.

Assessment	All Patients	Non-Oral Frailty Group	Oral Frailty Group	*p* Value
Revised J-CHS, %				0.007
Normal	21.6	25	13.3	
Pre-frailty	51	61.1	26.7	
Frailty	27.5	13.9	60	
FRAIL scale, %				0.022
Normal	9.8	13.9	0	
Pre-frailty	56.9	63.9	40	
Frailty	33.3	22.2	60	
				
AWGS2019, %				0.46
Normal	88.2	91.7	80	
Sarcopenia	3.9	2.8	6.7	
Severe sarcopenia	7.8	5.6	13.3	
				
SARC-CalF, %				0.047
Normal	80.4	88.9	60	
Sarcopenia	19.6	11.1	40	
				
MNA-SF, %				0.475
Normal	7.8	5.6	13.3	
Risk of malnutrition	49	47.2	53.3	
Malnutrition	43.1	47.2	33.3	
				
MUST, %				0.728
Low risk	64.7	61.1	73.3	
Moderate risk	23.5	25	20	
High risk	11.8	13.9	6.7	
				
GLIM, %				0.746
Normal	66.7	63.9	73.3	
Malnutrition	33.3	36.1	26.7	

AWGS2019, Asian Working Group for Sarcopenia in 2019; GLIM, Global Leadership Initiative on Malnutrition; J-CHS, Japanese version of the Cardiovascular Health Study; MNA-SF, Short-Form Mini-Nutritional Assessment; MUST, Malnutrition Universal Screening Tool; SARC-CalF, Screening Tool for Sarcopenia Combined with Calf Circumference.

**Table 4:** One-year changes in values for body indicators.

Variable	All Patients	Non-Oral Frailty Group	Oral Frailty Group	*p* Value
SMI, kg/m^2^	−0.02 ± 0.78	0.14 ± 0.65	−0.41 ± 0.93	0.018
CC, cm	0.26 ± 1.81	0.75 ± 1.54	−0.91 ± 1.92	0.002
Grip strength, kg	0.44 ± 4.05	0.90 ± 3.90	−0.68 ± 4.32	0.207
Gait speed, m/s	−0.03 ± 0.15	−0.02 ± 0.15	−0.09 ± 0.11	0.127
Weight, kg	−0.01 ± 3.24	1.18 ± 2.75	−2.87 ± 2.49	<0.001
BMI, kg/m^2^	−0.04 ± 1.22	0.40 ± 1.01	−1.10 ± 1.00	<0.001

BMI, body mass index; CC, calf circumference; SMI, skeletal muscle index.

**Table 5:** GLM analyses of associations between the OFI-8 risk category and 1-year changes in body indicators.

Outcome	Adjusted Mean Difference	SE	95% CI		*p* Value
			Lower	Upper	
SMI, kg/m^2^	–0.382	0.335	–1.060	0.293	0.261
CC, cm	–1.550	0.774	–3.110	0.006	0.051
Grip strength, kg	2.77	1.52	–0.285	5.83	0.075
Gait speed, m/s	0.046	0.159	–0.274	0.366	0.772
Weight, kg	–2.730	1.16	–5.060	–0.386	0.023
BMI, kg/m^2^	–1.090	0.439	–1.970	–0.203	0.017

BMI, body mass index; CC, calf circumference; CI, confidence interval; SE, standard error; SMI, skeletal muscle index.

## Data Availability

The data used in this study are available from the corresponding author due to ethical reasons.

## Supplementary Materials

The following supporting information can be downloaded at https://www.mdpi.com/article/10.3390/nu17121950/s1: Figure S1: Asian Working Group for Sarcopenia in 2019 (AWGS2019) algorithm for sarcopenia; Figure S2: Malnutrition Universal Screening Tool (MUST); Figure S3: Global Leadership Initiative on Malnutrition (GLIM) criteria; Table S1: Oral Frailty Index-8 (OFI-8) categories; Table S2: Revised Japanese version of the Cardiovascular Healthy Study (J-CHS) criteria; Table S3: FRAIL scale; Table S4: Screening Tool for Sarcopenia Combined with Calf Circumference (SARC-CalF); Table S5: Scoring on the Short-Form Mini-Nutritional Assessment (MNA-SF).
