# Supplementary material for: Association of Oral Frailty with Physical Frailty and Malnutrition in Patients on Peritoneal Dialysis
Source: Nutrients. 2025 Jun 6;17(12):1950. doi: 10.3390/nu17121950 (PMC12195954; doi:10.3390/nu17121950)
Supplement: Supplementary file 1 [file nutrients-17-01950-s001.zip › nutrients-3628584-supplementary.pdf]

Supplementary Table S1. Oral Frailty Index-8 categories

**Question**	**Applicable**	**Not applicable**
Do you have any difficulties eating tough foods compared with 6 months ago?	2	0
Have you choked on tea or soup recently?	2	0
Do you wear a denture?	2	0
Do you often experience dry mouth?	1	0
Do you go out less frequently than you did 6 months ago?	1	0
Can you eat foods as hard as squid jerky or pickled radish?	0	1
Do you brush your teeth at least twice a day?	0	1
Do you visit a dental clinic at least once a year?	0	1

**Total Score**	**Risk Category**
0–2	Low
3	Moderate
4–11	High

Supplementary Table S2. Revised Japanese version of the Cardiovascular Healthy Study (J-CHS) criteria

**Item**	**Evaluation criteria**	**Applicable**	**Not applicable**
Shrinking	Unintentional weight loss of ≥ 2 kg in the past 6 months	1	0
Weakness	Grip strength: men < 28.0 kg and < 18.0 kg for women	1	0
Exhaustion	Constant tiredness in the past 2 weeks	1	0
Slowness	Usual gait speed < 1.0 m/s	1	0
Low activity	1) Low levels of physical exercise < 1 day/week	1	0
	2) Regular physical activities <1 day/week		
	Applicable to both questions		

**Total Score**	**Risk category**
0 point	Normal
1–2 points	Pre-frailty
3–5 points	Frailty

Supplementary Table S3. FRAIL scale

Item	Questionnaire	Applicable	Not applicable
Fatigue	Are you fatigued?	1	0
Resistance	Cannot walk up one flight of stairs?	1	0
Ambulation	Cannot walk one block?	1	0
Illness	Do you have more than 5 illnesses?	1	0
Loss of weight	Have you lost more than 5% of your body weight in the last 6 months?	1	0

Total Score	Risk category
0 point	Robust
1–2 points	Pre-frailty
≥3 points	Frailty

Supplementary Table 4. Screening Tool for Sarcopenia Combined with Calf Circumference (SARC-CalF)

Item	Questionnaire	0	1	2
Strength	How much difficulty do you have in lifting and carrying 4-5 kg?	None	Some	A lot or anable
Assistance in walking	How much difficulty do you have walking across a room?	None	Some	A lot, use aids, or unable
Rise from a chair	How much difficulty do you have transferring from a chair or bed?	None	Some	A lot or unable without help
Climb stairs	How much difficulty do you have climbing a flight of 10 stairs?	None	Some	A lot or unable
Falls	How many times have you fallen in the past year?	None	Less than 3 falls	4 or more falls
Calf Circumference	If the value is below the cutoff of 34.0 cm in men and 33.0 cm in women, add 10 points to the score.			

Total score	Risk category
0	Robust
≥ 11	Suspicion of sarcopenia

Supplementary Table 5. Scoring on the Short-Form Mini-Nutritional Assessment (MNA-SF)

How much has your food intake decreased in the past 3 months because of loss of appetite, digestive problems, or chewing or swallowing difficulties?	Severe loss of appetite = 0	Moderate loss of appetite = 1	No loss of appetite = 2	
How much weight have you lost in the past 3 months?	≥3 kg = 0	Do not know = 1	1–3 kg = 2	No weight loss = 3
How far can you walk by yourself?	Bedridden or chairbound = 0	Capable of getting out of bed or a chair but cannot go out = 1	Capable of going out = 2	
Have you experienced psychological stress or an acute illness in the past 3 months?	Yes = 0		No = 2	
Neuropsychiatric problems	Severe dementia or depression = 0	Mild dementia = 1	No such problems = 2	
Body mass index, kg/m^2	<19 = 0	≥19, <21 = 1	≥21, <23 = 2	≥23 = 3

**Total Score**	**Evaluation Stage**
12–14	Normal, no need for further assessment
8–11	Risk of malnutrition
0–7	Malnutrition

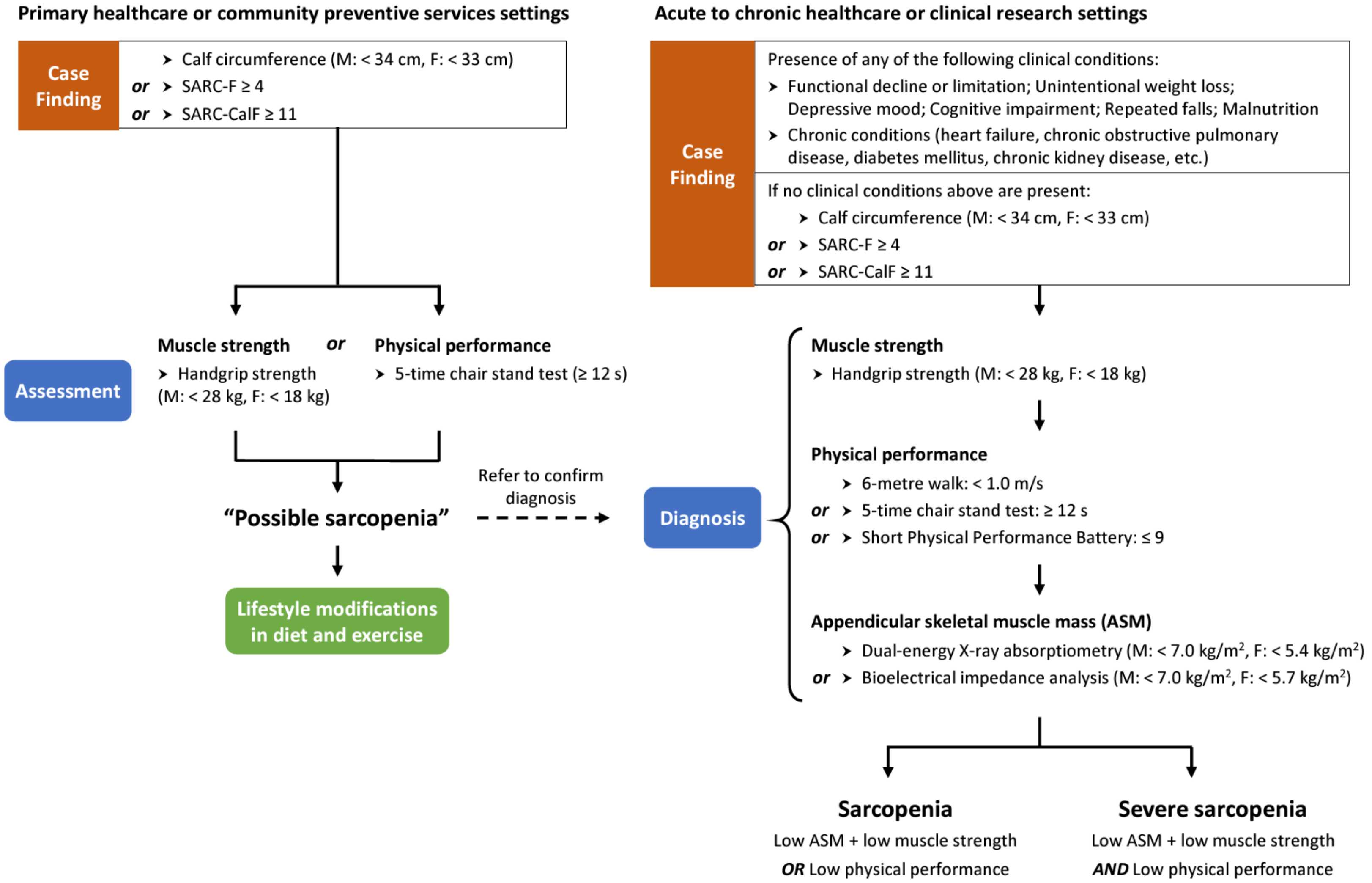

Low muscle mass + low muscle strength + low physical performance	Severe sarcopenia
Low muscle mass + low muscle strength or low physical performance	Sarcopenia
Neither of above	Normal

Supplementary Figure S1. Asian Working Group for Sarcopenia in 2019 (AWGS2019) algorithm for sarcopenia

BMI	Score
> 20	0
18.5-20	1
< 18.5	2

Weight loss in 3-6 months	Score
≤ 5%	0
5-10%	1
≥ 10%	2

Acute disease effect: add a score of 2
if there has been or is likely to be no or nutritional intake for > 5 days

Add scores

Total score	Risk category
0	Low risk
1	Medium risk
≥ 2	High risk

Supplementary Figure S2. Malnutrition Universal Screening Tool (MUST)

Risk screening:

Use validated screening tools, such as MUST, MNA-SF, NRS-2002, etc.

Diagnostic assessment:

Phenotypic criteria		
Weight loss	Low body mass index	Reduced muscle mass
>5% within the past 6 months, or >10% beyond 6 months	<18.5 if <70 years, or <20 if >70 years	Reduced by validated body composition measuring techniques

Etiologic criteria	
Reduced food intake or assimilation	Disease burden/Inflammatory condition
≤ 50% of energy requirements >1 week, or any reduction for >2 weeks, or any chronic gastrointestinal condition that adversely impacts food assimilation or absorption	Acute disease/injury, or chronic disease-related

Diagnosis:

Requires at least 1 phenotypic criterion and 1 etiologic criterion

Severity grading:

Weight loss	Low body mass index	Reduced muscle mass
>10% within the past 6 months, or >20% beyond 6 months	Severe deficit	Severe deficit

Supplementary Figure 3. Global Leadership Initiative on Malnutrition (GLIM) criteria
